# Shape-dependent cytotoxicity and cellular uptake of gold nanoparticles synthesized using green tea extract

**DOI:** 10.1186/s11671-019-2967-1

**Published:** 2019-04-11

**Authors:** You Jeong Lee, Eun-Young Ahn, Youmie Park

**Affiliations:** College of Pharmacy and Inje Institute of Pharmaceutical Sciences and Research, Inje University, 197 Inje-ro, Gimhae, Gyeongnam 50834 Republic of Korea

**Keywords:** Gold nanoparticles, Green tea extract, Nanospheres, Nanostars, Nanorods, Cytotoxicity, Cellular uptake

## Abstract

In the present report, three different shapes of chitosan-capped gold nanoparticles (nanospheres, nanostars, and nanorods) were synthesized to investigate the effects of shape on cytotoxicity and cellular uptake in cancer cells. Green tea extract was utilized as a reducing agent to reduce gold salts to gold nanospheres. Gold nanostars were prepared using an as-prepared nanosphere solution as a seed solution. Gold nanorods were synthesized using a conventional method. All three types of gold nanoparticles showed their characteristic surface plasmon resonance bands upon UV-visible spectrophotometry. In high-resolution transmission electron microscopy images, lattice structures were clearly observed in all three shapes, confirming the crystalline nature of the nanoparticles. All three colloidal solutions of gold nanoparticles retained colloidal stability in various solutions. To assess cytotoxicity, the 3-(4,5-dimethylthiazol-2-yl)-2,5-diphenyltetrazolium bromide (MTT) assay was performed on four cancer cell lines. The cytotoxicity was the highest in nanorods, followed by nanostars and finally nanospheres. The cellular uptake of gold nanoparticles in human hepatocyte carcinoma cells (HepG2) was measured, and the results followed the order nanospheres > nanorods > nanostars. The outcomes of the current study may assist in the shape design of gold nanoparticles for therapeutic applications as drug delivery vehicles in the field of nanomedicine.

## Introduction

Plants contain natural primary and secondary metabolites, including flavonoids, saponins, alkaloids, steroids, coumarins, tannins, phenols, terpenoids, carbohydrates, proteins, and amino acids. Recently, plant extracts have been utilized for the synthesis of nanomaterials, specifically metallic nanoparticles such as gold, silver, titanium oxide, copper, palladium, zinc oxide, and platinum nanoparticles [1]. Diverse phytochemicals actively participate in converting metal salts to metallic nanoparticles as reducing agents. In addition, plant extracts play a role as stabilizing agents to maintain the colloidal stability of metallic nanoparticles in solution. Chemical reducing agents are generally noxious and toxic to living organisms. By contrast, the use of plant extracts in the synthesis of metallic nanoparticles is green, eco-friendly, and sustainable. Various plant parts, such as stems, fruits, seeds, leaves, and flowers, are utilized to synthesize metallic nanoparticles [1, 2]. Extensive reviews have surveyed the green synthesis of gold nanoparticles (AuNPs) using plant extracts as reducing agents [2–4]. Using this green strategy, the synthetic step is performed in one step and in one pot. Furthermore, the process is simple, facile, cost-effective, and eco-friendly. The size, shape, and topography of AuNPs are dependent on the concentrations of gold salts and extracts, reaction time, reaction temperature, and solution pH. Spectroscopic and microscopic techniques are employed to characterize AuNPs, including UV-visible spectrophotometry, X-ray diffraction, Fourier-transform infrared spectroscopy (FT-IR), transmission electron microscopy (TEM), atomic force microscopy (AFM), scanning electron microscopy (SEM), and hydrodynamic size and zeta potential measurements. These green AuNPs are applied as catalysts, antioxidants, and antimicrobial and anticancer agents [5–8].

In the authors’ laboratory, plant extracts (*Artemisia capillaris*, *Leonurus japonicus*, *Polygala tenuifolia*, *Caesalpinia sappan*, *Bupleurum falcatum*, and *Garcinia mangostana*) have been used as reducing agents to green-synthesize both AuNPs and silver nanoparticles (AgNPs) [9–18]. The aerial part of *A. capillaris* was extracted and used for the synthesis of AgNPs and AuNPs [9, 15, 16]. The prepared AgNPs exerted excellent antibacterial activity against *Escherichia coli*, *Enterobacter cloacae*, *Pseudomonas aeruginosa*, *Klebsiella aerogenes*, and *Klebsiella oxytoca* compared with that of the extract alone [15]. This result indicated that the synergistic effect of combining AgNPs and the extract contributed to enhanced antibacterial activity. Interestingly, AgNPs synthesized using *A. capillaris* in the presence of cetyltrimethylammonium bromide exhibited antibacterial activity against methicillin-resistant *Staphylococcus aureus* [16]. Moreover, AuNPs synthesized using *A. capillaries* showed catalytic activity toward the 4-nitrophenol reduction reaction [9]. *L. japonicus* extract was utilized for the synthesis of AgNPs exhibiting remarkable enhancements in antibacterial activity [10]. We observed that antibacterial activity towards Gram-negative bacteria was greater than that against Gram-positive bacteria. The root extract of *P. tenuifolia* was also utilized for the synthesis of AuNPs and AgNPs [11, 17]. Enhanced anticoagulant activity and antibacterial activity were observed in AuNPs and AgNPs, respectively, synthesized using *P. tenuifolia* extract. Most interestingly, AgNPs synthesized using *C. sappan* extract showed effective antibacterial activity against methicillin-resistant *S. aureus* [18]. The extract of *G. mangostana* produced asymmetric dumbbell-shaped AgNPs with apoptotic effects [14].

Previous reports have examined the green synthesis of AuNPs and AgNPs using tea leaf extract [19–23]. Kamal and coworkers reported the successful synthesis of 25 nm-AgNPs [19]. In another report, spherical AgNPs measuring 20 to 90 nm were synthesized using tea leaf extract [20]. The synthesized AgNPs showed slight antibacterial activity against *E. coli*. Spherical AgNPs measuring 3.42~4.06 nm were also prepared by Loo and coworkers using tea leaf extract [21]. Vaseeharan and coworkers synthesized antibacterial AgNPs using tea leaf extract [22]. The synthesized AgNPs were effective against pathogenic *Vibrio harveyi* infection. Begum and coworkers used black tea leaf extract to synthesize both AuNPs and AgNPs [23]. To date, mostly spherical AgNPs have been synthesized using tea leaf extract.

In the present report, chitosan was used as a capping agent for AuNPs. Chitosan has been explored as a drug/gene delivery vehicle due to its high biocompatibility, low allergenicity, biodegradability, and low toxicity [24–26]. Chitosan originates from chitin, which is abundant in the exoskeletons of insects and crustaceans such as crabs, lobsters, and shrimp. Chitin is a polysaccharide composed of *N*-acetyl-D-glucosamine linked by β(1-4) glycosidic bonds. Chitosan can be obtained from chitin by a heterogeneous *N*-deacetylation process. Chitosan itself possesses antibacterial, antifungal, antitumor, and antioxidant activity [25]. As a capping agent, chitosan directly contacts AuNPs through an electrosteric mechanism [26]. Chitosan nanoparticles affect the mechanism of cellular uptake by A549 cells without modifying cytotoxicity [24]. Furthermore, the degree of deacetylation of chitosan is more influential than molecular weight on cellular uptake and cytotoxicity [24].

Most studies have focused on the synthesis of spherical AgNPs using tea leaf extract. Herein, green tea leaf extract was utilized to synthesize gold nanospheres and nanostars. The nanospheres were used as seeds for the seed-mediated synthesis of nanostars. For comparison, nanorods were synthesized by a common conventional method [27]. Three different shapes of AuNPs (nanospheres, nanostars, and nanorods) were capped with chitosan to increase their biocompatibility and colloidal stability. These AuNPs were characterized by UV-visible spectrophotometry, high-resolution transmission electron microscopy (HR-TEM), and FT-IR. Hydrodynamic size measurements performed by dynamic light scattering (DLS) and zeta potential measurements were conducted before and after capping with chitosan. Colloidal stability was assessed in salt solutions, buffer, and cell culture medium. To measure cytotoxicity, the 3-(4,5-dimethylthiazol-2-yl)-2,5-diphenyltetrazolium bromide (MTT) assay was applied to four cancer cells: AGS (human gastric adenocarcinoma cells), HeLa (human epithelial cervix adenocarcinoma cells), HepG2 (human hepatocyte carcinoma cells), and HT29 (human colorectal adenocarcinoma cells). Cellular uptake of AuNPs in HepG2 cells was quantitatively measured by inductively coupled plasma optical emission spectroscopy (ICP-OES) and laser ablation inductively coupled plasma mass spectrometry (LA-ICP-MS).

## Materials and methods

### Materials and instrumentation

Chloroauric acid trihydrate (HAuCl_4_·3H_2_O), cetyltrimethylammonium bromide (CTAB), chitosan (from shrimp shells, ≥ 75 % deacetylated), and 3-(4,5-dimethylthiazol-2-yl)-2,5-diphenyltetrazolium bromide were purchased from Sigma-Aldrich (St. Louis, MO, USA). All other reagents were of analytical grade. A Shimadzu UV-1800 or UV-2600 spectrophotometer was utilized to acquire UV-visible spectra in a quartz cuvette (Shimadzu Corporation, Kyoto, Japan). Hydrodynamic size measurements performed by DLS and zeta potential measurements were conducted using a NanoBrook 90Plus Zeta (Brookhaven Instruments Corporation, New York, USA). A Varian 640 IR was used to acquire FT-IR spectra (Agilent Technologies, Santa Clara, CA, USA); the sample measured was prepared by the KBr disc method. HR-TEM images were captured using a JEM-3010 operated at 300 kV (JEOL, Tokyo, Japan); the sample was loaded onto a carbon-coated copper grid (carbon type-B, 300 mesh, Ted Pella, Redding, CA, USA) and allowed to oven-dry at 37 °C for 24 h. For sonication, a model WUC-A22H was used (Daihan Scientific Co. LTD., Seoul, Republic of Korea). A Centrifuge 5424R (Eppendorf AG, Hamburg, Germany) and an FD8518 (IlshinBioBase Co. LTD., Gyeonggi, Republic of Korea) were used for centrifugation and freeze-drying, respectively. For the cellular uptake of AuNPs, an Optima 8300 ICP-OES (PerkinElmer, Waltham, MA, USA) and a J200 Tandem LA-ICP-MS (Applied Spectra, Fremont, CA, USA) were utilized.

### Preparation of green tea extract

The Institute of Hadong Green Tea (Hadong, Gyeongnam, Republic of Korea) kindly provided a gift of dried green tea leaves. A blender was used to prepare powders of the dried leaves. Extraction was performed by mixing deionized water (2 L) and powdered leaves (200 g). Extraction was allowed to proceed for 1 h by sonication at ambient temperature with three repetitions. Whatman filter papers were used to filter water fractions to remove insoluble materials. Then, the filtrate was centrifuged (3,000*g* force, 18 °C, 25 min), and the supernatant was collected. The collected supernatant was syringe-filtered, and the filtrate was freeze-dried. The freeze-dried material was dissolved in deionized water to create a stock solution with a final concentration of 2 % (*w/v*) for the green synthesis described in the following section.

### Synthesis of gold nanospheres using extract

**Fig. 1:**
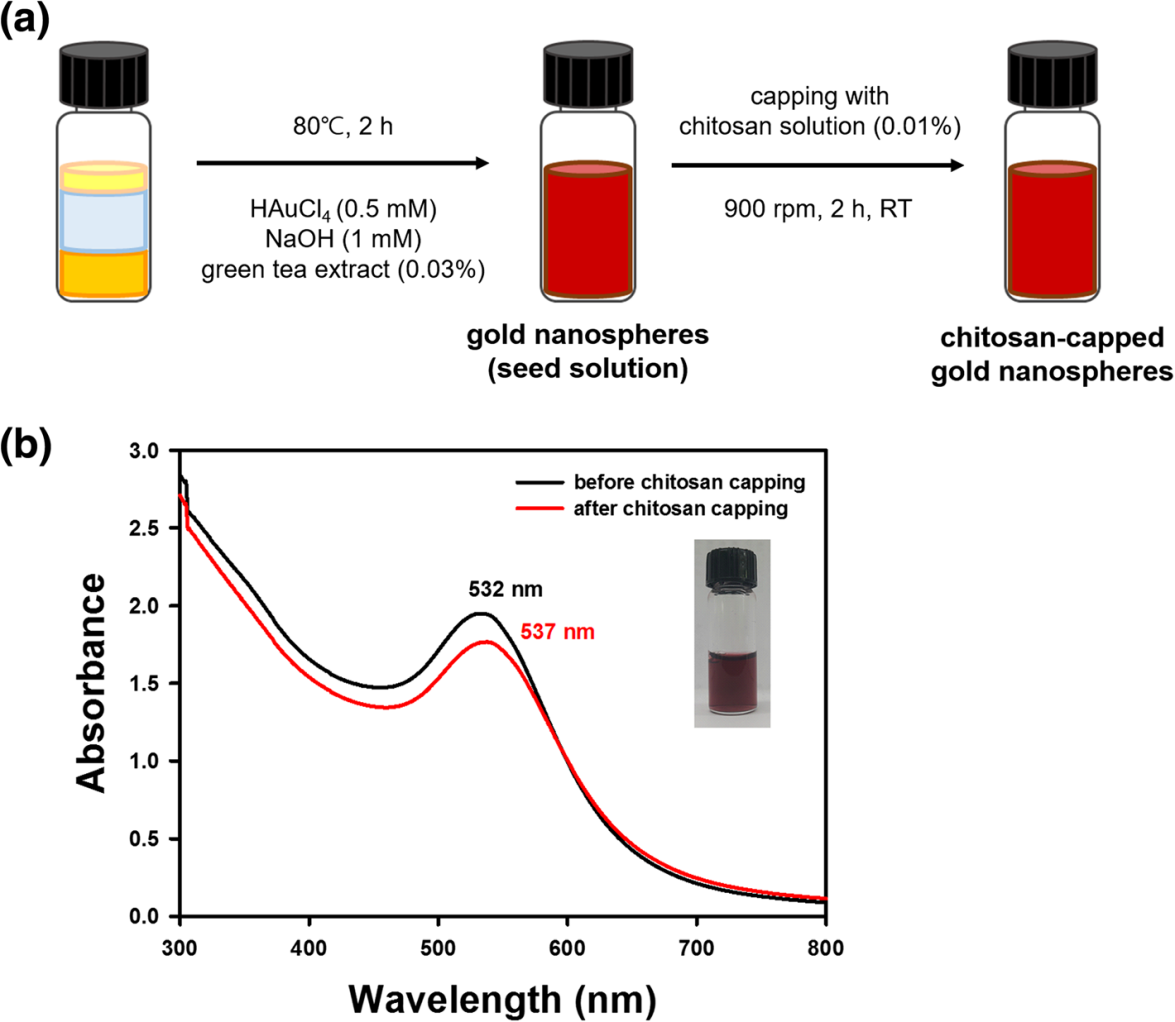
Synthesis of gold nanospheres. **a** A schematic diagram of the synthesis process and **b** UV-visible spectra of nanospheres before and after chitosan capping. A digital photograph shows nanospheres immediately after synthesis

The nanosphere synthesis process is illustrated in Fig. 1a. The stock solution described in the previous section was used for synthesis. In a glass vial, the extract (final concentration of 0.03 %) and chloroauric acid trihydrate (final concentration of 0.5 mM) were mixed, and sodium hydroxide (final concentration of 1 mM) was added. Deionized water was added to make a final volume of 2 mL. Oven incubation was performed in a dry oven at 80 °C for 2 h. The surface plasmon resonance (SPR) of the nanospheres was monitored by acquiring UV-visible spectra over the range of 300~800 nm. The nanospheres were also used as seeds for the synthesis of nanostars described in the following section.

### Synthesis of gold nanostars

**Fig. 2:**
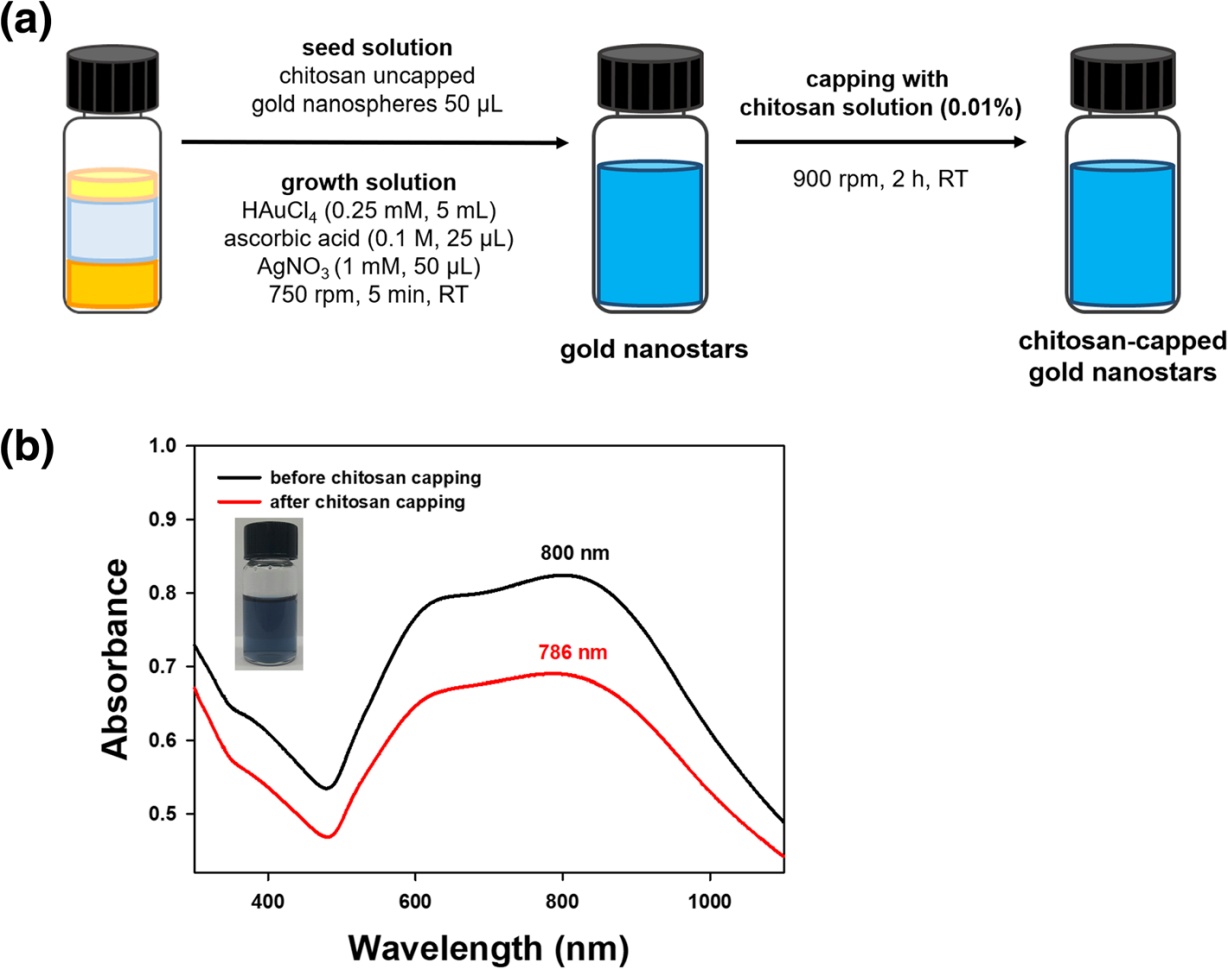
Synthesis of gold nanostars. **a** A schematic diagram of the synthesis process and **b** UV-visible spectra of nanostars before and after chitosan capping. A digital photograph shows nanostars immediately after synthesis

The nanostar synthesis process is illustrated in Fig. 2a. The nanospheres (50 μL) synthesized as described in the previous section were stirred (750 rpm) with a magnetic bar on a hot plate at ambient temperature. Chloroauric acid trihydrate (0.25 mM, 5 mL) was added to this solution. After 15 s, two solutions were added simultaneously: silver nitrate (1 mM, 50 μL) and ascorbic acid (freshly prepared, 100 mM, 25 μL). Then, the mixture was stirred at 750 rpm for 5 min. UV-visible spectra were acquired over the range of 300~1100 nm.

### Synthesis of gold nanorods

**Fig. 3:**
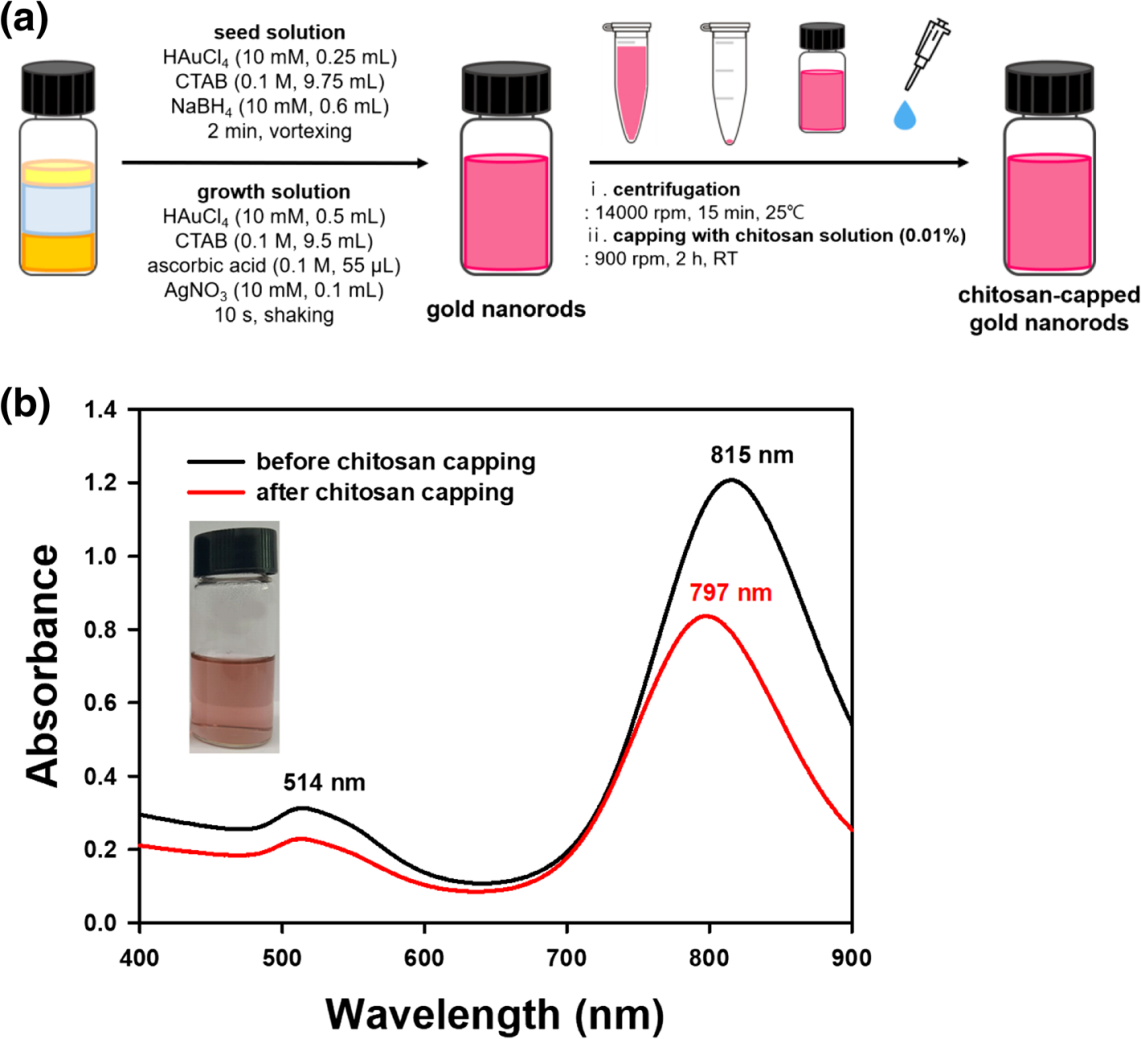
Synthesis of gold nanorods. **a** A schematic diagram of the synthesis process and **b** UV-visible spectra of nanorods before and after chitosan capping. A digital photograph shows nanorods immediately after synthesis

The nanorod synthesis process is illustrated in Fig. 3a. For the synthesis of gold nanorods, seed-mediated synthesis was performed according to a previous report with minor modifications [27]. The growth solution was prepared as follows. In a 20 mL glass vial, chloroauric acid trihydrate (10 mM, 500 μL) and cetyltrimethylammonium bromide (CTAB, 100 mM, 9.5 mL) were mixed. The solution was yellowish brown. Next, ascorbic acid (freshly prepared, 100 mM, 55 μL) was added, and this solution was stirred until it turned from yellowish brown to colorless. Silver nitrate (10 mM, 100 μL) was added and shaken for 10 s. This final solution was labeled a growth solution. The seed solution was synthesized as follows. In a 20 mL glass vial, chloroauric acid trihydrate (10 mM, 250 μL) and CTAB (100 mM, 9.75 mL) were mixed. Then, ice-cold, freshly prepared sodium borohydride (10 mM, 600 μL) was added and vortexed for 2 min. The solution was labeled a seed solution. The seed solution (12 μL) was mixed with a growth solution synthesized previously. UV-visible spectra were acquired over the range of 400~900 nm.

### Chitosan capping of nanospheres, nanostars, and nanorods

Chitosan capping was employed to increase the colloidal stability and biocompatibility of AuNPs. Chitosan was dissolved in 1 % acetic acid, and the final concentration was adjusted to 0.01 %. Then, sonication was performed to completely dissolve chitosan; this solution was used as a stock solution for chitosan capping in the following procedure. Both nanospheres and nanostars that were previously synthesized were capped with the chitosan stock solution (0.01 %). The chitosan stock solution (30 %, *v/v*) was mixed with the AuNP solution (70 %, *v/v*). The mixture was stirred at 900 rpm for 2 h to complete chitosan capping. For nanorods, excess CTAB was used, and thus, centrifugation (14,000 rpm, 15 min, 25 °C) was performed to remove CTAB. The nanorods were recovered from the pellet, and the chitosan capping was conducted as mentioned above. Then, UV-visible spectra were acquired.

### Assessment of colloid stability

The colloidal stability of AuNPs is important for in vitro and in vivo applications. The three types of AuNPs with chitosan capping and nanospheres without chitosan capping were evaluated for colloidal stability in the following solutions: deionized water, 5 % bovine serum albumin (BSA), 5 % NaCl, PBS (pH 7.4), Dulbecco’s modified Eagle’s medium (DMEM), and full medium. The full medium was DMEM containing 10 % fetal bovine serum (FBS). One milliliter of each type of AuNP solution was mixed with the testing solution as mentioned above (0.5 mL). The mixture was incubated at 25 °C for 30 min, and UV-visible spectra were acquired.

### Cell culture and cytotoxicity

The following cancer cell lines were purchased from the Korean Cell Line Bank (Seoul, Republic of Korea): AGS, HeLa, HepG2, and HT29. The MTT assay was performed to evaluate the in vitro cytotoxicity of AuNPs. DMEM containing sodium pyruvate was utilized. Cell culture medium contained 10 % fetal bovine serum, 2 mM L-glutamine, 1 % penicillin (100 units/mL), and streptomycin (100 units/mL). Cells were cultured in 100 mm culture dishes and maintained at approximately 70 % confluence. Before cell culture, the AuNPs of three different shapes were subjected to vacuum evaporation to obtain a final Au concentration of 5 mM. On 96-well plates, the cells were seeded at a density of 5.0 × 10^3^ cells/well, and incubation was conducted for 24 h in an oven at 37 °C under a CO_2_ (5 %) atmosphere. Next, five different concentrations of AuNPs (500 μM, 250 μM, 125 μM, 62.5 μM, and 31.25 μM) were treated and incubated in the oven for another 24 h at 37 °C under a CO_2_ (5 %) atmosphere. Next, MTT reagent (5 μL, 5 % in deionized water) was added and incubated in a 37 °C oven under a CO_2_ (5 %) atmosphere for an additional 3 h. The absorbance was measured at 570 nm using a fluorescence multi-detection reader (Synergy HT, Bio Tek Instruments, Winooski, VT, USA). Untreated cells were utilized as a control.

### Cellular uptake

The cellular uptake of each type of AuNP was quantitatively measured using HepG2 cells. On 24-well plates, the cells were seeded at a density of 5.0 × 10^4^ cells/well, and incubation was conducted for 24 h in an oven at 37 °C under a CO_2_ (5 %) atmosphere. Next, 5 μM (final concentration) of each type of AuNP solution was treated and incubated in the oven for an additional 24 h at 37 °C under a CO_2_ (5 %) atmosphere. After incubation, the solution containing excess AuNPs was removed, and the cells were treated with trypsin. The Au concentration in the trypsinized cells was quantitatively measured by ICP-OES and LA-ICP-MS, which provided the concentration of Au uptake in the cells. The control was 5 μM of the original colloidal solution of each type of AuNP, which was also analyzed by ICP-OES and LA-ICP-MS to obtain the Au concentration.

## Results and discussion

### UV-visible spectra

UV-visible spectra are commonly acquired to confirm the synthesis of AuNPs. The characteristic SPR of AuNPs can be observed over the visible-near-infrared wavelength range. Moreover, the capping of AuNPs generally induces either a bathochromic (or red) or a hypsochromic (or blue) shift. Furthermore, a hypochromic shift is generally induced together with red and blue shifts. As shown in Fig. 1b, UV-visible spectra were monitored over a range of 300~800 nm. Nanospheres showed a characteristic SPR of 532 nm with a deep burgundy color. After the chitosan capping of nanospheres, the maximum SPR was red-shifted to 537 nm together with a hypochromic shift (Fig. 1b). A wide range of SPR wavelengths (600~800 nm) with a dark blue solution were observed for the nanostars (Fig. 2b). The chitosan capping of the nanostars showed a hypochromic shift, where the absorbance was lower than that of AuNPs without chitosan capping (Fig. 2b). Nanorods showed two distinct SPR wavelengths of 514 nm and 815 nm, with the formation of a light-pink solution (Fig. 3b). The chitosan capping of nanorods induced a blue shift to 797 nm together with a hypochromic shift (Fig. 3b). Considering all these results, the capping of AuNPs with chitosan changed the SPR wavelength and induced shifts. Careful examination of the UV-visible spectra demonstrated that the three types of AuNPs were successfully capped with chitosan.

### Hydrodynamic size and zeta potentials

Next, the hydrodynamic size and zeta potentials were measured; the results are shown in Table 1. Without chitosan capping, the hydrodynamic sizes of nanospheres and nanostars were 28.4 and 97.8 nm, respectively. The hydrodynamic size of the nanorods was not measured because the hydrodynamic size was well adjusted to the spherical shapes of the nanoparticles. With chitosan capping, the hydrodynamic size was increased to 190.7 nm for nanospheres and to 123.9 nm for nanostars. Chitosan capping was confirmed by an increase in hydrodynamic size. The change in zeta potentials also reflected chitosan capping on the surface of AuNPs. Chitosan is a positively charged polysaccharide; thus, chitosan capping resulted in positive zeta potentials for all three types of AuNPs. For the nanospheres, the zeta potential was altered from − 12.73 to 42.28 mV. The zeta potential of the nanostars was altered from − 42.46 to 47.44 nm. In the case of the nanorods, CTAB (a cationic surfactant) was utilized for synthesis. Thus, the original zeta potential was 27.96 mV without capping. The chitosan capping of nanorods increased the zeta potential to 33.23 nm. Therefore, the change in zeta potentials from negative to positive values for the nanospheres and nanostars clearly indicated successful capping with chitosan. Furthermore, the zeta potential of the nanorods increased, suggesting that the surface was capped with chitosan.

**Table 1:** Hydrodynamic size and zeta potentials of nanoparticles with and without chitosan capping

		Nanospheres	Nanostars	Nanorods
Hydrodynamic size (nm)	Without chitosan capping	28.4	97.8	–^a^
	With chitosan capping	190.7	123.9	–^a^
Zeta potentials (*ζ*, mV)	Without chitosan capping	− 12.73	− 42.46	27.96
	With chitosan capping	42.28	47.44	33.23

^a^The hydrodynamic size of nanorods was not measured

### HR-TEM images

**Fig. 4:**
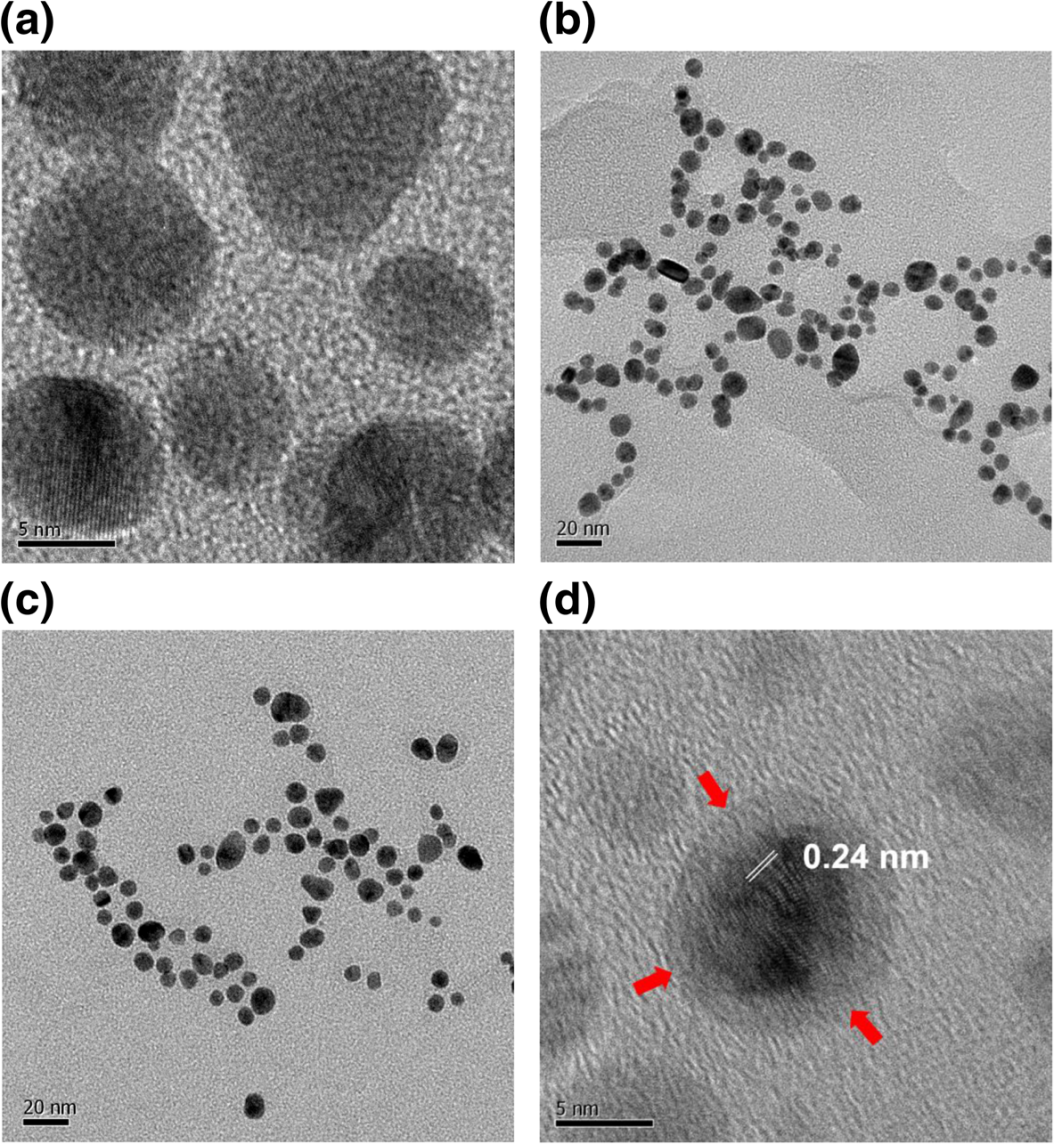
HR-TEM images of gold nanospheres. The scale bars represent **a** 5 nm, **b** 20 nm, **c** 20 nm, and **d** 5 nm. Images **a** and **b** were obtained without chitosan capping, and **c** and **d** were obtained with chitosan capping. The distance between neighboring lattices was measured to be 0.24 nm. Red arrows indicate the chitosan layer after capping

**Fig. 5:**
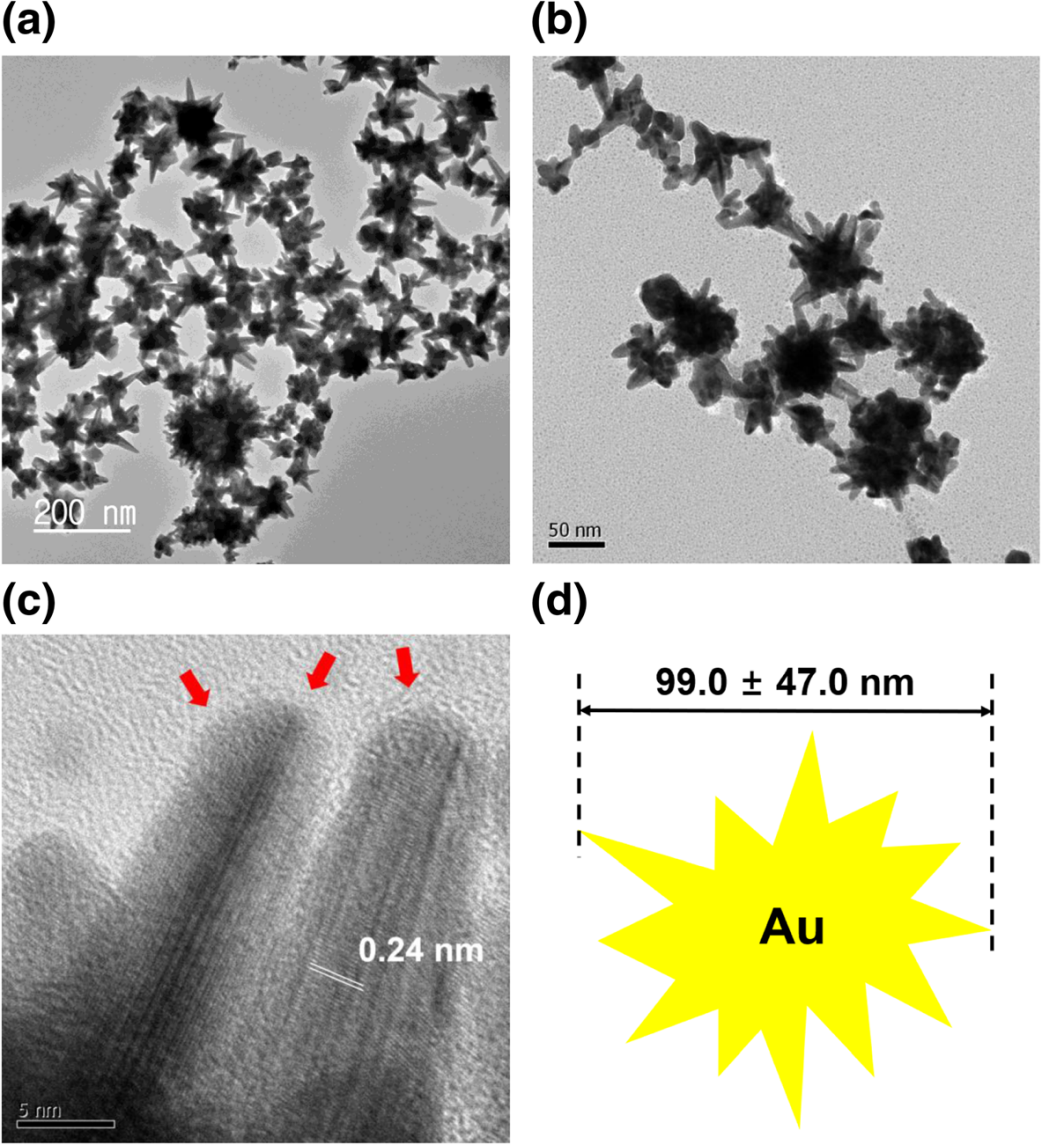
HR-TEM images of gold nanostars. The scale bars represent **a** 200 nm, **b** 50 nm, and **c** 5 nm. **d** A schematic representation of nanostars with an average size of 99.0 ± 47.0 nm. Image **a** was obtained without chitosan capping, and **b** and **c** were obtained with chitosan capping. The distance between neighboring lattices was measured to be 0.24 nm. Red arrows indicate the chitosan layer after capping

**Fig. 6:**
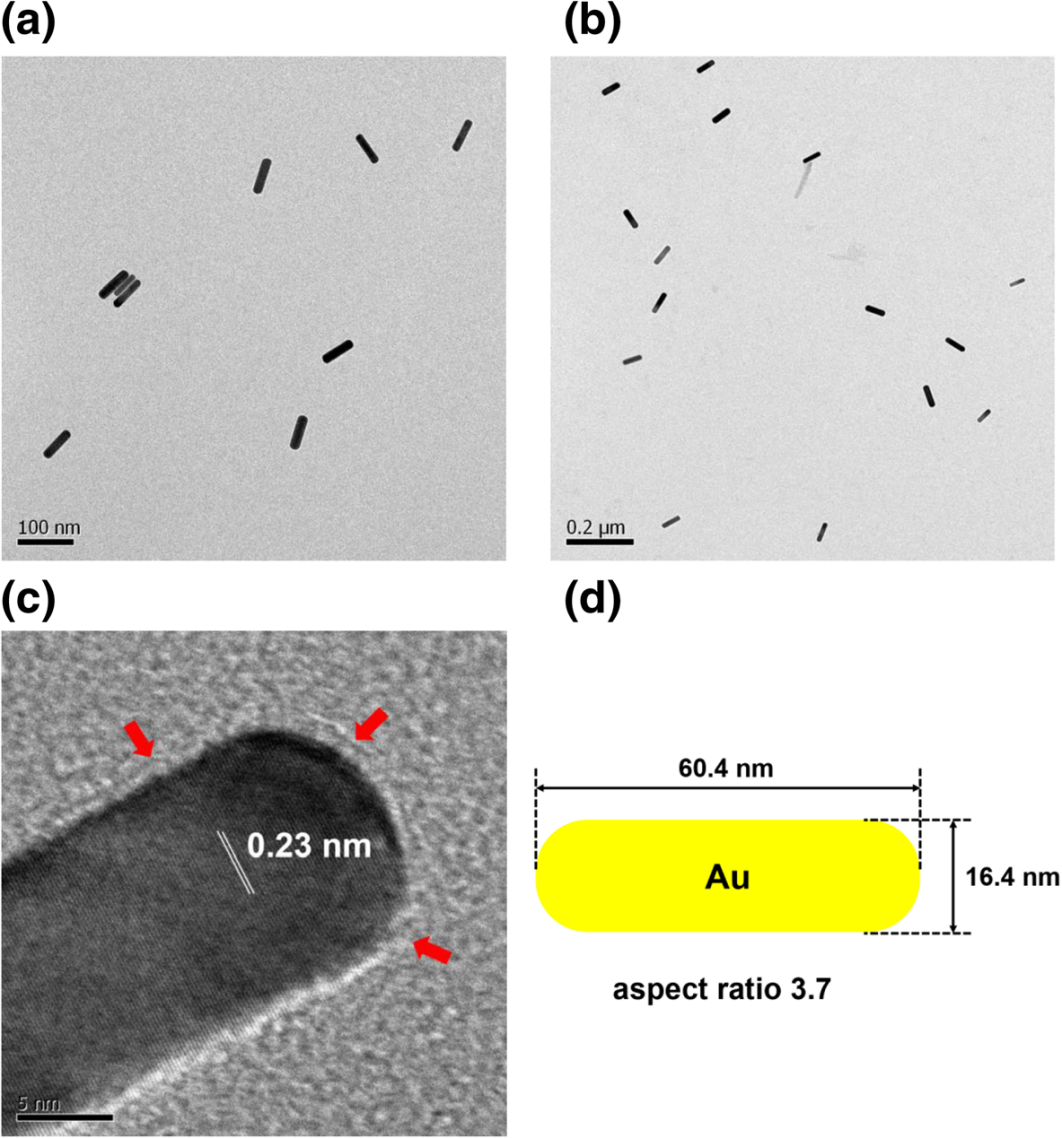
HR-TEM images of gold nanorods. The scale bars represent **a** 100 nm, **b** 200 nm, and **c** 5 nm. **d** A schematic representation of nanorods with an average length and width of 60.4 nm and 16.4 nm, respectively. Image **a** was obtained without chitosan capping, and **b** and **c** were obtained with chitosan capping. The distance between neighboring lattices was measured to be 0.23 nm. Red arrows indicate the chitosan layer after capping

Microscopy is crucial for nanoparticle research, providing essential information about the size and shape of nanoparticles. Microscopy tools deliver a range of detailed information, such as the dispersion state, two- and three-dimensional morphology and topography, and relative softness/hardness of materials. The nanospheres measured 8.7 ± 1.7 nm, based on the average of 75 randomly selected discrete nanoparticles in HR-TEM images (Fig. 4). The most abundant size was 8~9 nm (29.3 %), followed by 9~10 nm (12.0 %). With chitosan capping, the shape of the nanospheres was conserved without any change in shape (Fig. 4c, d). The crystalline nature of the particles was clearly indicated by the lattice structures shown in Fig. 4d. The distance between neighboring lattices was measured to be 0.24 nm (Fig. 4d). Furthermore, the chitosan layer was also visualized, as indicated by the red arrows in Fig. 4d. Nanostars were visualized by HR-TEM, as shown in Fig. 5. The nanostars measured 99.0 ± 47.0 nm, representing the average of 19 discrete nanoparticles. The lattice structure is indicated in a magnified image (Fig. 5c). As shown, the distance between neighboring lattices was measured to be 0.24 nm (Fig. 5c). The red arrows in Fig. 5c indicate the chitosan layer. Nanorods were visualized as shown in Fig. 6. The average particle length and width were 60.4 nm and 16.4 nm, respectively, according to measurements made for 28 particles. The aspect ratio, defined as particle length divided by particle width, was 3.7. The lattice structure is shown in Fig. 6c, confirming the crystalline structure of the nanorods. The distance between neighboring lattices was measured to be 0.23 nm (Fig. 6c). The red arrows indicate the chitosan layer (Fig. 6c).

### FT-IR spectra

**Fig. 7:**
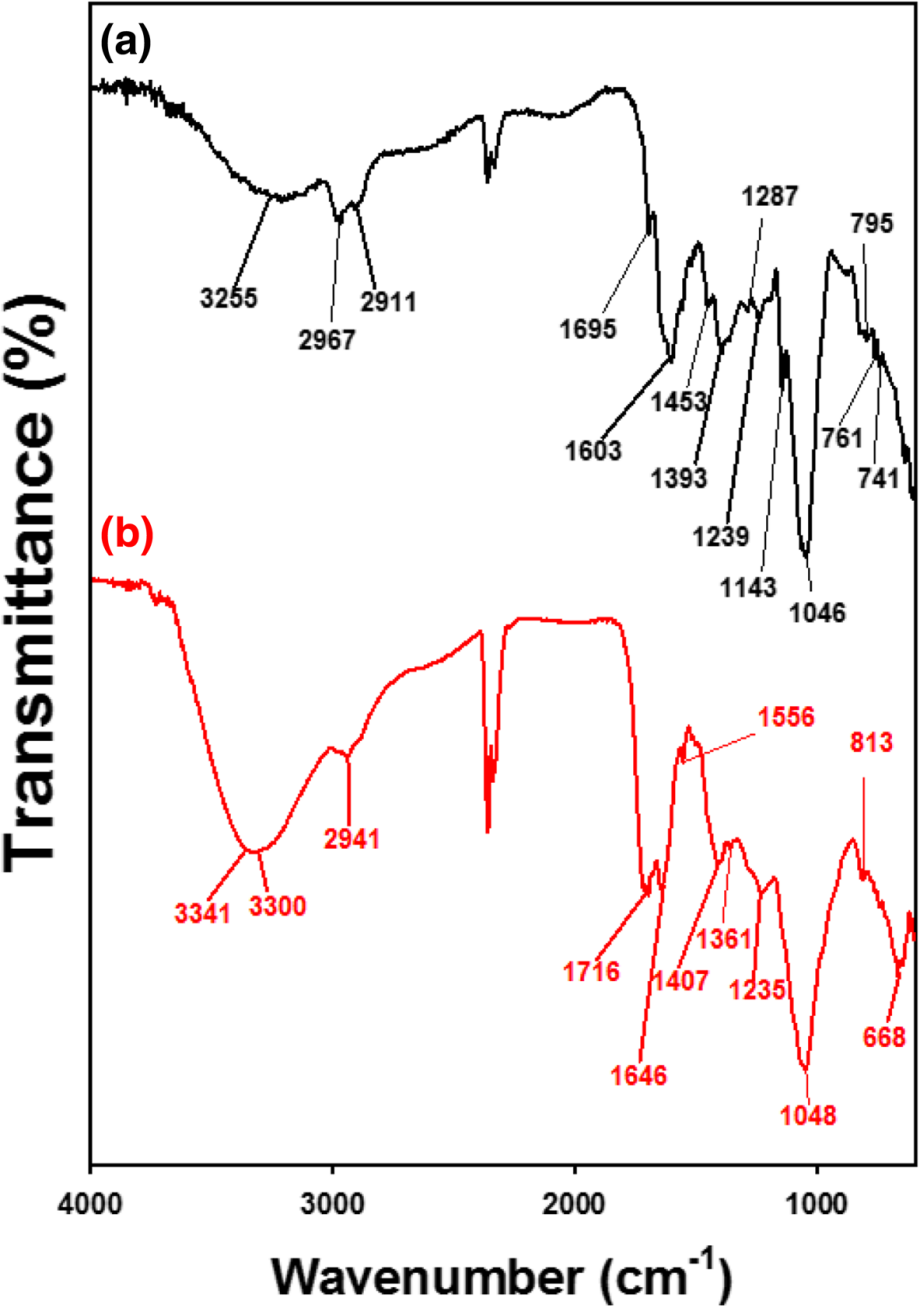
FT-IR spectra of **a** green tea extract used for synthesis and **b** gold nanospheres

Green tea contains diverse primary and secondary metabolites. Specifically, the major constituents of green tea are polyphenols, including epigallocatechin-3-gallate, (-)-epicatechin-3-gallate, (-)-epigallocatechin, and (-)-epicatechin [28]. FT-IR spectra were acquired to obtain information about functional groups that contributed to the synthesis of AuNPs. We compared the FT-IR spectrum of nanospheres with the spectrum of the extract (Fig. 7). The major functional group that was most likely involved in the reduction reaction of Au salts was –OH. In the extract, –OH functional groups appeared at 3255 cm^−1^ (Fig. 7a). Upon synthesis, this peak was shifted to a higher wavenumber at 3300~3341 cm^−1^. This result demonstrated that –OH functional groups originated from polyphenols oxidized to C=O while reducing Au salts to AuNPs. Remarkably, the appearance of C=O functional groups at 1716 cm^−1^ in nanospheres clearly supported the oxidation of –OH functional groups during synthesis (Fig. 7b).

### Assessment of colloidal stability under various solutions

**Fig. 8:**
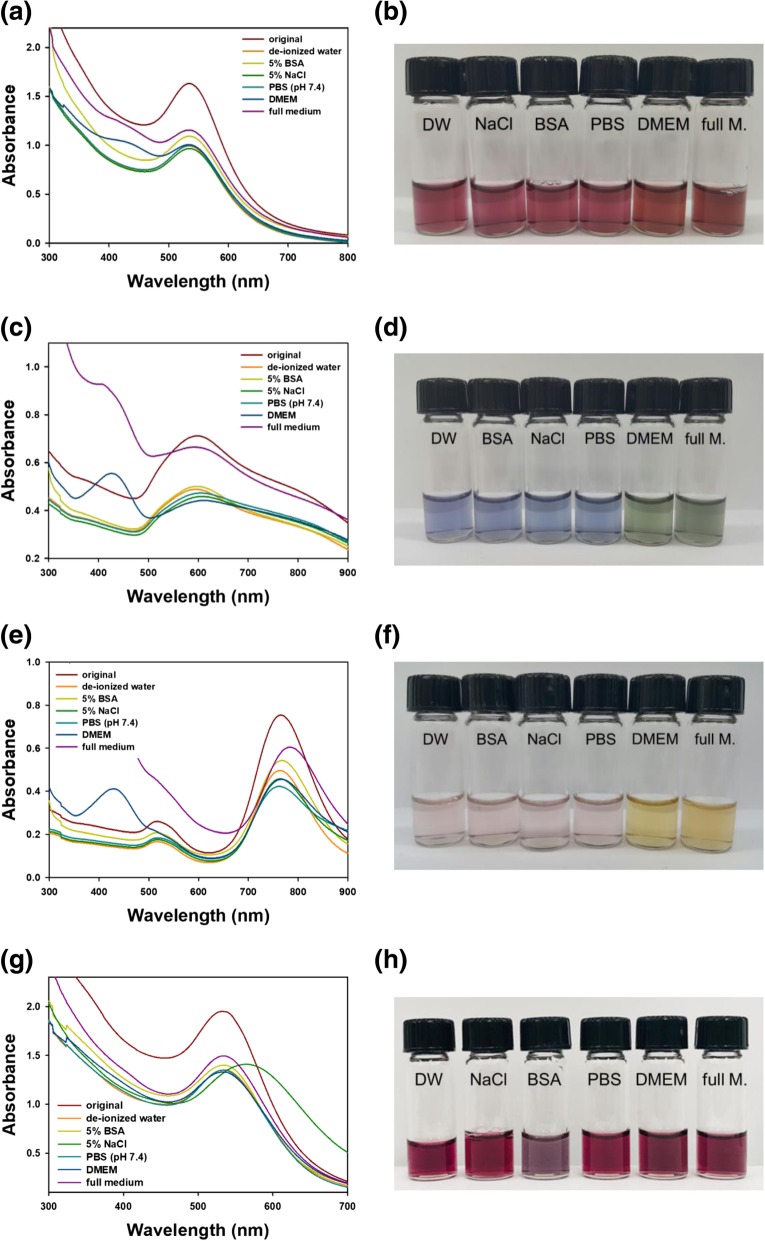
Assessment of colloidal stability. **a** and **b** nanospheres with chitosan capping, **c** and **d** nanostars with chitosan capping, **e** and **f** nanorods with chitosan capping, **g** and **h** nanospheres without chitosan capping. DW represents de-ionized water

The colloidal stability of nanoparticles is an important concern for diagnostic and therapeutic applications. Six different solutions were tested for colloidal stability: (i) deionized water, (ii) NaCl (5 %), (iii) PBS (pH 7.4), (iv) BSA (5 %), (v) DMEM, and (vi) full medium (DMEM with 10 % FBS). After mixing each type of AuNPs with the testing solution, UV-visible spectra were acquired; the results are shown in Table 2 and Fig. 8. A hypochromic shift together with a slight red or blue shift was observed for all three types of AuNPs in the UV-visible spectra (Fig. 8a, c, and e). The shape of the spectra was retained, and no aggregation of the colloidal solution was observed (Fig. 8b, d, and f). This result demonstrated that the colloidal stability was quite well retained in the abovementioned testing solutions.

**Table 2:** Colloidal stability of AuNPs with chitosan capping in different solutions

Nanospheres				Nanostars			Nanorods		
	Maximum SPR (nm)	Absorbance	%	Maximum SPR (nm)	Absorbance	%	Maximum SPR (nm)	Absorbance	%
Original	537	1.768	100	599	0.712	100	765	0.754	100
Deionized water	534	0.993	56.2	597	0.489	68.7	764	0.496	65.8
5 % BSA	535	1.093	61.8	597	0.500	70.2	767	0.543	72.0
5 % NaCl	535	0.966	54.6	608	0.458	64.3	765	0.457	60.6
PBS (pH 7.4)	535	1.000	56.6	604	0.473	66.4	763	0.422	56.0
DMEM	534	1.006	56.9	610	0.441	61.9	766	0.455	60.3
Full medium	533	1.155	65.3	592	0.665	93.4	783	0.605	80.2

In Table 2, the hypochromic shift is expressed as the percentage of retained absorbance, with the absorbance of the original solution set to 100 %. In all three AuNPs, colloidal stability was best retained in full medium, which was used for the subsequent cytotoxicity experiments: nanospheres (65.3 %), nanostars (93.4 %), and nanorods (80.2 %). The protein solution, BSA (5 %), also provided reasonable colloidal stability: nanospheres (61.8 %), nanostars (70.2 %), and nanorods (72.0 %). These results indicated that the proteins that cover the nanoparticles have further beneficial effects on the colloidal stability of the AuNPs together with the chitosan capping. The colloidal stability of the nanospheres without chitosan capping was also assessed (Fig. 8g, h). All solutions induced a hypochromic shift. Among the tested solutions, only the NaCl (5 %) solution showed a large red shift along with a hypochromic shift.

### Cytotoxicity

**Fig. 9:**
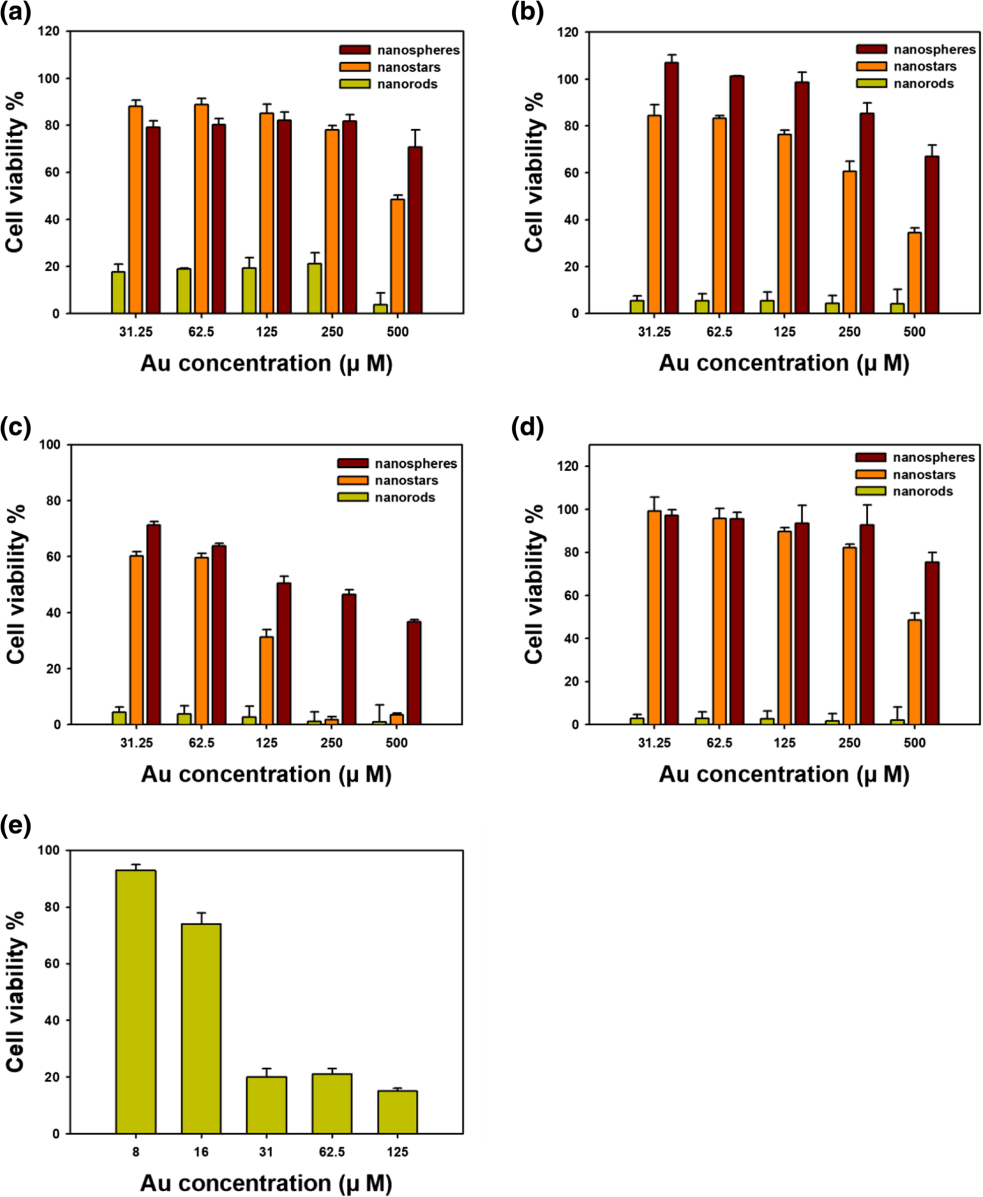
Cytotoxicity assessed by MTT assay (31.25~500 μM Au concentration). **a** AGS, **b** HeLa, **c** HepG2, and **d** HT29. **e** Low Au concentrations (8~125 μM) were evaluated on HepG2 cells

An MTT assay was performed to measure cytotoxicity against four cancer cell types (Fig. 9): AGS, HeLa, HepG2, and HT29. The cytotoxicity of all three types of AuNPs was dependent on the Au concentration. Among the four cell types, the highest cytotoxicity was observed for HepG2 cells. Furthermore, nanorods showed the highest toxicity against the four cell types, followed by nanostars and finally by nanospheres. Specifically, nanorods showed a very high toxicity; thus, the cytotoxicity of nanorods containing a low range of Au concentrations was also evaluated (Fig. 9e). At concentrations as low as 8 μM Au, nanorods showed concentration-dependent cytotoxicity. Against HepG2 cells, the IC_50_ value was 127.1 μM Au for nanospheres, 81.8 μM Au for nanostars, and 22.7 μM Au for nanorods. Thus, nanorods were the most cytotoxic, followed by nanostars, and nanospheres were the least cytotoxic against HepG2 cells.

It has been reported that the cytotoxicity of AuNPs is affected by size, shape, and surface charge [29, 30]. Favi and co-workers investigated the cytotoxicity of Au nanospheres (61 nm) and Au nanostars (34 nm) against two types of cells (human skin fibroblasts and rat fat pad endothelial cells) [31]. In both cell types, a lethal concentration was observed at 40 μg/mL for nanospheres and at 400 μg/mL for nanostars. Their results suggested that nanospheres were more cytotoxic than nanostars, suggesting that size, shape, and surface chemistry are most likely influential to the cytotoxicity of AuNPs. Woźniak and co-workers reported the cytotoxicity of AuNPs with diverse shapes against both HeLa and HEK293T (human embryonic kidney cells), namely, nanospheres (~ 10 nm), nanoflowers (~ 370 nm), nanorods (~ 41 nm), nanoprisms (~ 160 nm), and nanostars (~ 240 nm) [30]. Interestingly, nanospheres and nanorods were more cytotoxic than nanoflowers, nanoprisms, and nanostars. The authors explained that the small size of the nanoparticles and the aggregation process were the main driving forces for the cytotoxicity of nanospheres and nanorods in their work. Indeed, many studies have addressed the size effect of AuNPs on cytotoxicity [32, 33]. As AuNPs become smaller, their uptake by cells increases. This higher uptake results in a higher concentration of AuNPs in the cell, which leads to higher cytotoxicity against cells. However, a low concentration of AuNPs in the cell also shows high cytotoxicity [34]. The concentration of AuNPs in the cell affects cytotoxicity; however, it is vital to consider and understand the characteristics of AuNPs. The cytotoxicity of AuNPs of two different shapes (nanospheres 43 nm, nanorods 38 × 17 nm) was evaluated in epithelial cells by Tarantola and co-workers [35]. Nanospheres were determined to be more cytotoxic than nanorods. Furthermore, nanospheres induced a dysfunction in epithelial cell membranes, which was measured by electric-cell substrate impedance sensing [35]. As previously discussed, many studies are currently investigating the cytotoxicity of different shapes of AuNPs in various cells. Although the shapes of AuNPs may remain the same, many factors still affect the cytotoxicity of AuNPs. When assessing cytotoxicity, factors including size, shape, physicochemical surface properties, concentration, exposure time, and cell type should also be considered [34, 36–38]. In addition to the characteristics of AuNPs, cytotoxic mechanisms including disruption of the cell membrane, oxidative stress, destruction of the cytoskeleton, and loss of mitochondrial function are also important [38, 39]. Currently, autophagy and lysosomal dysfunction are emerging as explanations of the cytotoxicity of nanomaterials [40]. In lysosomes, nanomaterials induce cytotoxicity by lysosomal-iron-mediated oxidative stress and the release of cathepsins and other associated lysosomal hydrolases, which causes mitochondrial dysfunction and cell death. In the current report, nanorods were the most cytotoxic against the four types of cancer cells tested. Thus, our future work will examine the detailed mechanisms of cytotoxicity.

### Cellular uptake on HepG2 cells

**Fig. 10:**
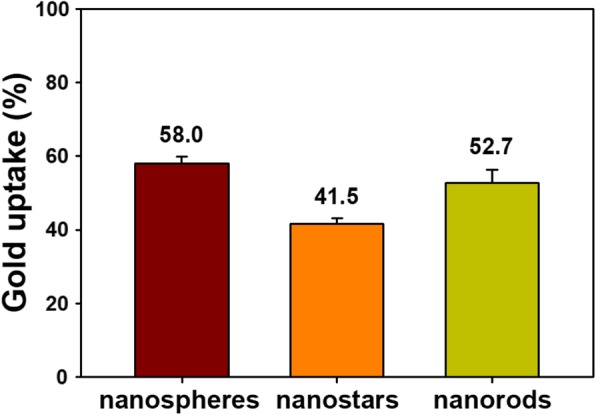
Cellular uptake by HepG2 cells

HepG2 cells showed the highest cytotoxicity among the four types of cancer cells; accordingly, we selected this cell type for evaluating cellular uptake. Two instruments, ICP-OES and LA-ICP-MS, were used to quantitatively analyze the concentration of Au in cells, and the results are shown in Fig. 10. A Au concentration of 5 μM was used for evaluating cellular uptake because no toxicity was observed at this concentration among the four types of cancer cells. The uptake was the highest for nanospheres (58.0 %), followed by nanorods (52.7 %) and nanostars (41.5 %). As indicated in the previous section, the cytotoxicity was dependent on particle shape (nanorods > nanostars > nanospheres). However, the order of cellular uptake (nanospheres > nanorods > nanostars) did not match the order of cytotoxicity. The cellular uptake of nanospheres was the highest; however, the cytotoxicity of these particles was the lowest. These results suggest that high cellular uptake does not always induce high cytotoxicity. As mentioned previously, diverse factors including size, shape, physicochemical surface properties, concentration, exposure time, and cell type are important in influencing cytotoxicity.

The different degrees of uptake of the three types of AuNPs (41.5~58.0 %) possibly depend on the competition between wrapping (i.e., how a membrane encloses a nanoparticle) under a thermodynamic driving force and receptor diffusion kinetics [41, 42]. Chithrani and Chan compared the cellular uptake of transferrin-coated Au nanospheres and Au nanorods by HeLa cells [42]. At the same size (i.e., the same value for the nanosphere diameter and nanorod width), nanospheres showed higher uptake than did nanorods. This result is consistent with our observations in the current report of higher uptake of nanospheres compared with nanorods. For nanorod uptake, width is more important than length, and an increasing aspect ratio decreases the uptake rate [42]. The size of the nanostars was 99.0 ± 47.0 nm, making them the largest particles among the three types of AuNPs. For large nanoparticles (> 50 nm), slow receptor diffusion kinetics lead to short wrapping times [42]. Thus, the cellular uptake of large nanoparticles is low, i.e., nanostars in the current report. Chitosan was used for capping the AuNPs to increase their colloidal stability and biocompatibility. Chitosan capping can also play a role in the cellular uptake of AuNPs by interacting with receptors on the cell surface. A detailed mechanistic study is necessary to elucidate this issue.

## Conclusion

The continued development of nanotechnology requires elaborate shape and size designs of nanoparticles for successful applications, including as drug delivery carriers or vehicles for biologically active compounds such as anticancer agents. Green tea extract was used as a green reducing agent for the synthesis of Au nanospheres and nanostars. Interestingly, the cytotoxicity of nanorods was higher than that of nanospheres and nanostars, while nanospheres showed the lowest cytotoxicity against four types of cancer cells. Cellular uptake by HepG2 cells was most likely dependent on shape and size; nanospheres showed the highest uptake by the cells, whereas nanostars showed the lowest uptake. The optimization of size and shape together with surface modification and functionalization will lead to the development of nanoparticles for future use in nanomedicine.

## Abbreviations

AFM: Atomic force microscopy
AgNPs: Silver nanoparticles
AGS: Human gastric adenocarcinoma cells
AuNPs: Gold nanoparticles
CTAB: Cetyltrimethylammonium bromide
DLS: Dynamic light scattering
DMEM: Dulbecco’s modified Eagle’s medium
FBS: Fetal bovine serum
FT-IR: Fourier-transform infrared spectroscopy
HeLa: Human epithelial cervix adenocarcinoma cells
HepG2: Human hepatocyte carcinoma cells
HR-TEM: High-resolution transmission electron microscopy
HT29: Human colorectal adenocarcinoma cells
ICP-OES: Inductively coupled plasma optical emission spectroscopy
LA-ICP-MS: Laser ablation inductively coupled plasma mass spectrometry
SEM: Scanning electron microscopy
SPR: Surface plasmon resonance
